# Supplementary material for: DNA Methylation Malleability and Dysregulation in Cancer Progression: Understanding the Role of PARP1
Source: Biomolecules. 2022 Mar 8;12(3):417. doi: 10.3390/biom12030417 (PMC8946700; doi:10.3390/biom12030417)
Supplement: Supplementary file 1 [file biomolecules-12-00417-s001.zip › biomolecules-1559534-supplementary.pdf]

Table S1. Dysregulation of DNMTs/TET in different types of cancer

Protein	Cancers with Dysregulated DNMTs/TETs	References
DNMT1	Acute myeloid leukemia	[1]
	Breast cancer	[2-4]
	Colorectal cancer	[5,6]
	Gastric cancer	[7]
	Lung cancer	[8,9]
	Pancreatic cancer	[10]
	Pituitary adenoma	[11]
	Thyroid cancer	[12]
DNMT2	Colorectal cancer	[6]
	Stomach cancer	[6]
DNMT3A	Acute myeloid leukemia	[13,14]
	Pituitary adenoma	[11]
	Vulvar squamous cell carcinoma	[15]
DNMT3B	Bladder cancer	[16]
	Breast cancer	[4,17]
	Hepatocellular carcinoma	[18]
	Lung cancer	[19]
	Ovarian cancer	[20]
	Stomach cancer	[6]
TETs	Acute myeloid leukemia	[21]
	Bladder cancer	[22]
	Chronic lymphocytic leukemia	[23]
	Colorectal carcinomas	[24,25]
	Endometrioid carcinoma	[26]
	Lung cancer	[27-29]
	Skin cancer	[27]

References

1. Yu, J.; Peng, Y.; Wu, L.C.; Xie, Z.; Deng, Y.; Hughes, T.; He, S.; Mo, X.; Chiu, M.; Wang, Q.E.; et al. Curcumin down-regulates DNA methyltransferase 1 and plays an anti-leukemic role in acute myeloid leukemia. *PLoS ONE* **2013**, *8*, e55934. https://doi.org/10.1371/journal.pone.0055934.
2. Li, Y.; Meeran, S.M.; Patel, S.N.; Chen, H.; Hardy, T.M.; Tollefsbol, T.O. Epigenetic reactivation of estrogen receptor-α (ERα) by genistein enhances hormonal therapy sensitivity in ERα-negative breast cancer. *Mol. Cancer* **2013**, *12*, 9. https://doi.org/10.1186/1476-4598-12-9.
3. Wang, N.; Wang, Z.; Wang, Y.; Xie, X.; Shen, J.; Peng, C.; You, J.; Peng, F.; Tang, H.; Guan, X.; et al. Dietary compound isoliquiritigenin prevents mammary carcinogenesis by inhibiting breast cancer stem cells through WIF1 demethylation. *Oncotarget* **2015**, *6*, 9854–9876. https://doi.org/10.18632/oncotarget.3396.
4. Qin, W.; Zhang, K.; Clarke, K.; Weiland, T.; Sauter, E.R. Methylation and miRNA effects of resveratrol on mammary tumors vs. normal tissue. *Nutr. Cancer* **2014**, *66*, 270–277. https://doi.org/10.1080/01635581.2014.868910.

5. Kanai, Y.; Ushijima, S.; Nakanishi, Y.; Sakamoto, M.; Hirohashi, S. Mutation of the DNA methyltransferase (DNMT) 1 gene in human colorectal cancers. *Cancer Lett.* **2003**, *192*, 75–82. https://doi.org/10.1016/s0304-3835(02)00689-4.
6. Kanai, Y.; Ushijima, S.; Kondo, Y.; Nakanishi, Y.; Hirohashi, S. DNA methyltransferase expression and DNA methylation of CPG islands and peri-centromeric satellite regions in human colorectal and stomach cancers. *Int. J. Cancer* **2001**, *91*, 205–212. https://doi.org/10.1002/1097-0215(200002)9999:9999<::aid-ijc1040>3.0.co;2-2.
7. Etoh, T.; Kanai, Y.; Ushijima, S.; Nakagawa, T.; Nakanishi, Y.; Sasako, M.; Kitano, S.; Hirohashi, S. Increased DNA methyltransferase 1 (DNMT1) protein expression correlates significantly with poorer tumor differentiation and frequent DNA hypermethylation of multiple CpG islands in gastric cancers. *Am. J. Pathol.* **2004**, *164*, 689–699.
8. Lin, R.-K.; Wu, C.-Y.; Chang, J.-W.; Juan, L.-J.; Hsu, H.-S.; Chen, C.-Y.; Lu, Y.-Y.; Tang, Y.-A.; Yang, Y.-C.; Yang, P.-C. Dysregulation of p53/Sp1 control leads to DNA methyltransferase-1 overexpression in lung cancer. *Cancer Res.* **2010**, *70*, 5807–5817.
9. Chen, Y.; Tang, Q.; Xiao, Q.; Yang, L.; Hann, S.S. Targeting EP4 downstream c-Jun through ERK1/2-mediated reduction of DNMT1 reveals novel mechanism of solamargine-inhibited growth of lung cancer cells. *J. Cell Mol. Med.* **2017**, *21*, 222–233. https://doi.org/10.1111/jcmm.12958.
10. Huang, L.; Hu, B.; Ni, J.; Wu, J.; Jiang, W.; Chen, C.; Yang, L.; Zeng, Y.; Wan, R.; Hu, G.; et al. Transcriptional repression of SOCS3 mediated by IL-6/STAT3 signaling via DNMT1 promotes pancreatic cancer growth and metastasis. *J. Exp. Clin. Cancer Res.* **2016**, *35*, 27. https://doi.org/10.1186/s13046-016-0301-7.
11. Ma, H.S.; Wang, E.L.; Xu, W.F.; Yamada, S.; Yoshimoto, K.; Qian, Z.R.; Shi, L.; Liu, L.L.; Li, X.H. Overexpression of DNA (Cytosine-5)-Methyltransferase 1 (DNMT1) And DNA (Cytosine-5)-Methyltransferase 3A (DNMT3A) Is Associated with Aggressive Behavior and Hypermethylation of Tumor Suppressor Genes in Human Pituitary Adenomas. *Med. Sci. Monit.* **2018**, *24*, 4841–4850. https://doi.org/10.12659/MSM.910608.
12. Zhang, Y.; Sun, B.; Huang, Z.; Zhao, D.-W.; Zeng, Q. Shikonin inhibites migration and invasion of thyroid cancer cells by downregulating DNMT1. *Med. Sci. Monit. Int. Med. J. Exp. Clin. Res.* **2018**, *24*, 661.
13. Yan, X.-J.; Xu, J.; Gu, Z.-H.; Pan, C.-M.; Lu, G.; Shen, Y.; Shi, J.-Y.; Zhu, Y.-M.; Tang, L.; Zhang, X.-W. Exome sequencing identifies somatic mutations of DNA methyltransferase gene DNMT3A in acute monocytic leukemia. *Nat. Genet.* **2011**, *43*, 309–315.
14. Zhou, L.; Fu, L.; Lv, N.; Liu, J.; Li, Y.; Chen, X.; Xu, Q.; Chen, G.; Pang, B.; Wang, L. Methylation-associated silencing of BASP1 contributes to leukemogenesis in t (8; 21) acute myeloid leukemia. *Exp. Mol. Med.* **2018**, *50*, 1–8.
15. Leonard, S.; Pereira, M.; Fox, R.; Gordon, N.; Yap, J.; Kehoe, S.; Luesley, D.; Woodman, C.; Ganesan, R. Over-expression of DNMT3A predicts the risk of recurrent vulvar squamous cell carcinomas. *Gynecol. Oncol.* **2016**, *143*, 414–420.
16. Qiu, W.; Lin, J.; Zhu, Y.; Zhang, J.; Zeng, L.; Su, M.; Tian, Y. Kaempferol modulates DNA methylation and downregulates DNMT3B in bladder cancer. *Cell. Physiol. Biochem.* **2017**, *41*, 1325–1335.
17. Roll, J.D.; Rivenbark, A.G.; Jones, W.D.; Coleman, W.B. DNMT3b overexpression contributes to a hypermethylator phenotype in human breast cancer cell lines. *Mol. Cancer* **2008**, *7*, 1–14.
18. Lai, S.-C.; Su, Y.-T.; Chi, C.-C.; Kuo, Y.-C.; Lee, K.-F.; Wu, Y.-C.; Lan, P.-C.; Yang, M.-H.; Chang, T.-S.; Huang, Y.-H. DNMT3b/OCT4 expression confers sorafenib resistance and poor prognosis of hepatocellular carcinoma through IL-6/STAT3 regulation. *J. Exp. Clin. Cancer Res.* **2019**, *38*, 1–18.
19. Kuck, D.; Caulfield, T.; Lyko, F.; Medina-Franco, J.L. Nanaomycin A selectively inhibits DNMT3B and reactivates silenced tumor suppressor genes in human cancer cells. *Mol. Cancer Ther.* **2010**, *9*, 3015–3023.
20. Zhao, L.; Shou, H.; Chen, L.; Gao, W.; Fang, C.; Zhang, P. Effects of ginsenoside Rg3 on epigenetic modification in ovarian cancer cells. *Oncol. Rep.* **2019**, *41*, 3209–3218.
21. Dolnik, A.; Engelmann, J.C.; Scharfenberger-Schmeer, M.; Mauch, J.; Kelkenberg-Schade, S.; Haldemann, B.; Fries, T.; Kronke, J.; Kuhn, M.W.; Paschka, P.; et al. Commonly altered genomic regions in acute myeloid leukemia are enriched for somatic mutations involved in chromatin remodeling and splicing. *Blood* **2012**, *120*, e83–e92. https://doi.org/10.1182/blood-2011-12-401471.
22. Gui, Y.; Guo, G.; Huang, Y.; Hu, X.; Tang, A.; Gao, S.; Wu, R.; Chen, C.; Li, X.; Zhou, L.; et al. Frequent mutations of chromatin remodeling genes in transitional cell carcinoma of the bladder. *Nat. Genet.* **2011**, *43*, 875–878. https://doi.org/10.1038/ng.907.
23. Quesada, V.; Conde, L.; Villamor, N.; Ordonez, G.R.; Jares, P.; Bassaganyas, L.; Ramsay, A.J.; Bea, S.; Pinyol, M.; Martinez-Trillos, A.; et al. Exome sequencing identifies recurrent mutations of the splicing factor SF3B1 gene in chronic lymphocytic leukemia. *Nat. Genet.* **2011**, *44*, 47–52. https://doi.org/10.1038/ng.1032.
24. Cancer Genome Atlas Network. Comprehensive molecular characterization of human colon and rectal cancer. *Nature* **2012**, *487*, 330–337. https://doi.org/10.1038/nature11252.

25. Seshagiri, S.; Stawiski, E.W.; Durinck, S.; Modrusan, Z.; Storm, E.E.; Conboy, C.B.; Chaudhuri, S.; Guan, Y.; Janakiraman, V.; Jaiswal, B.S.; et al. Recurrent R-spondin fusions in colon cancer. *Nature* **2012**, *488*, 660–664. https://doi.org/10.1038/nature11282.
26. Scourzic, L.; Mouly, E.; Bernard, O.A. TET proteins and the control of cytosine demethylation in cancer. *Genome Med.* **2015**, *7*, 9. https://doi.org/10.1186/s13073-015-0134-6.
27. Kan, Z.; Jaiswal, B.S.; Stinson, J.; Janakiraman, V.; Bhatt, D.; Stern, H.M.; Yue, P.; Haverty, P.M.; Bourgon, R.; Zheng, J.; et al. Diverse somatic mutation patterns and pathway alterations in human cancers. *Nature* **2010**, *466*, 869–873. https://doi.org/10.1038/nature09208.
28. Imielinski, M.; Berger, A.H.; Hammerman, P.S.; Hernandez, B.; Pugh, T.J.; Hodis, E.; Cho, J.; Suh, J.; Capelletti, M.; Sivachenko, A.; et al. Mapping the hallmarks of lung adenocarcinoma with massively parallel sequencing. *Cell* **2012**, *150*, 1107–1120. https://doi.org/10.1016/j.cell.2012.08.029.
29. Seo, J.S.; Ju, Y.S.; Lee, W.C.; Shin, J.Y.; Lee, J.K.; Bleazard, T.; Lee, J.; Jung, Y.J.; Kim, J.O.; Shin, J.Y.; et al. The transcriptional landscape and mutational profile of lung adenocarcinoma. *Genome Res* **2012**, *22*, 2109–2119. https://doi.org/10.1101/gr.145144.112
